# Ten years of data on small mammal species in Doñana (SW Spain): 2011–2021

**DOI:** 10.3897/BDJ.13.e160994

**Published:** 2025-10-27

**Authors:** Francisco Carro, Xosé Pardavila, Álvaro Martín, Carlos Caro, Isidro Román, Ramón-Casimiro Soriguer, Luis Santamaría, Javier Bustamante, Rocío Márquez-Ferrando, Ricardo Díaz-Delgado

**Affiliations:** 1 Estación Biológica de Doñana (CSIC), Sevilla, Spain Estación Biológica de Doñana (CSIC) Sevilla Spain; 2 Junta de Andalucía, Sevilla, Spain Junta de Andalucía Sevilla Spain; 3 ICTS-Reserva Biológica de Doñana, Almonte, Spain ICTS-Reserva Biológica de Doñana Almonte Spain

**Keywords:** capture-mark and recapture methods, Life Adaptamed Project, Long-Term Ecological Research, rodents, sampling event, Sherman live traps, shrews, Unique Scientific and Technical Infrastructure

## Abstract

**Background:**

The long-term monitoring of the small mammal community in Doñana (SW Spain) was initiated in 2011 as part of the European Long-Term Ecological Research (eLTER) project, of which Doñana is a Long-Term Socio-Ecological Research (LTSER) Platform. The main objective of this action is to collect time-series data on the abundance and distribution of the different small mammal species present in Doñana. Since 2017, this action has been included in the monitoring programme of the Integrated Scientific and Technical Infrastructure of the Doñana Biological Reserve (ICTS-Doñana) and in the LIFE Adaptamed project. The dataset includes information obtained from five representative habitats of Doñana and six sites sampled twice a year (spring and autumn). Some of them have been part of an experiment with different vegetation treatments applied. The sampling is based on capture and recapture methods using Sherman live traps. Information on sex, age and weight of each individual is also included. Here, we present data from 2011 to 2021, which could be useful for analyses of abundance, diversity and distribution patterns of the following species: white-toothed shrew (*Crocidura russula*), black rat (*Rattus rattus*), garden dormouse (*Eliomys quercinus*), Algerian mouse (*Mus spretus*) and wood mouse (*Apodemus sylvaticus*). This information can be used to increase the ecological knowledge and improve management plans for the conservation of the species in Mediterranean ecosystems.

**New information:**

This is the first published version of the standardised dataset of long-term monitoring of small mammals (Soricomorpha and Rodentia) in Doñana.

## Introduction

Small mammals (rodents and soricomorphs) are a diverse and species-rich group with similar ecological functions due to their small size (Barnett and Dutton 1995), contributing to ecosystem functioning and functional diversity (Paniccia et al. 2022). They are globally distributed from polar to tropical regions (Pagel et al. 1991). They are r-strategic species (i.e. short-lived individuals with small body size, high number of offspring and small home ranges) and occupy an intermediate position in food webs (as dispersers of seeds and fungal spores, pollinators, predators of seeds and invertebrates, prey of raptors, reptiles and mammalian carnivores) (Golley et al. 1975, Grant and French 1980). Their presence in ecosystems also benefits vegetation and soil regeneration (Lacher et al. 2019), which, along with the above reasons, makes them useful bioindicators (Pearce and Venier 2005, Blois et al. 2010). Their numbers can fluctuate between seasons and years (Golley et al. 1975, Lin and Batzli 2001) and show rapid responses to environmental factors, land use and predation (Pearce and Venier 2005, Blois et al. 2010, Osman et al. 2022, Torre and Balčiauskas 2023). In order to promote effective conservation actions for small mammal species, it is necessary to obtain more basic ecological knowledge by monitoring their populations to obtain a data series on abundance, spatial distribution, movements, age structures, sex ratio and body size.

**Figure 1.**
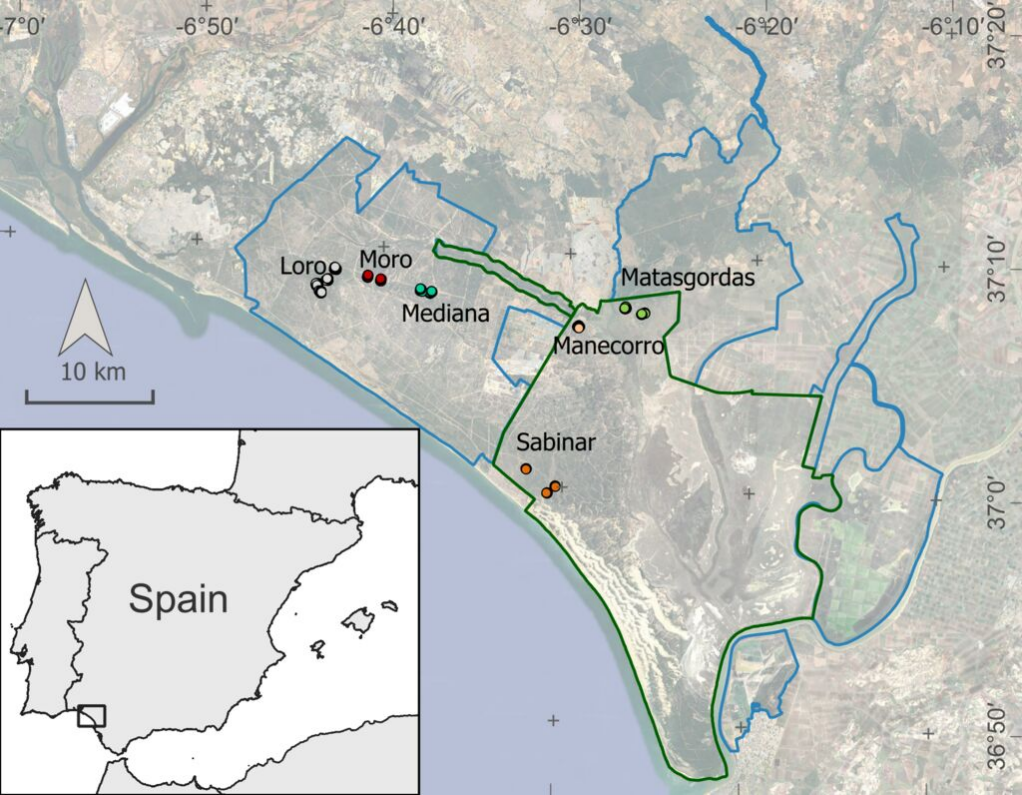
Geographic location of the six localities and plots surveyed in Doñana Natural (blue boundaries) and National (green boundaries) Park. The protected area is located in south-western Spain.

In Doñana National Park (DNP), south-western Spain (Fig. 1), the small mammal community has been sampled discontinuously over the last few decades (Marfil et al. 2009, Moreno and Rouco 2013, Moreno et al. 2016, Santoro et al. 2016). Eight species have been recorded: wood mouse (*Apodemus sylvaticus*), Algerian mouse (*Mus spretus*), garden dormouse (*Elyomis quercinus*), black rat (*Rattus rattus*), Mediterranean pine vole (*Microtus duodecimcostatus*), Western hedgehog (*Erinaceus europaeus*), white-toothed shrew (*Crocidura russula*) and pygmy white-toothed shrew (*Suncus etruscus*) (Palomo et al. 2007, Moreno and Rouco 2013). Different trends in population size have been observed across species, presumably associated with climate change and predation (Santoro et al. 2016). In the whole protected area of Doñana (DNP and the contiguous Natural Park), ecosystems suffer from negative impacts caused by several factors, such as extreme temperatures in summer, droughts, overgrazing, pests, forest fires and the lowering of groundwater levels due to agricultural intensification (Green et al. 2018, Green et al. 2024). Here, we present a long-term monitoring dataset of small mammals in Doñana (2011–2021), with the aim of increasing the abundant and biological data for this group. This information may be useful for future research projects and biodiversity conservation plans in Doñana and other Mediterranean ecosystems.

## Project description

### Title

European Long-Term Ecological Research (eLTER).

Long-term Doñana monitoring by the Unique Scientific and Technical Infrastructure of Doñana Biological Reserve (ICTS-Doñana) (ref.: 202030E286).

Life Adaptamed (Protection of Key Ecosystem Services by Adaptive Management of Climate Change Endangered Mediterranean Socioecosystems) (ref.: LIFE14 CCA/ES/000612).

### Personnel

Francisco Carro, Xosé Pardavilla, Álvaro Martín, Carlos Caro, Isidro Román, Ramón Soriguer Escofet, Luis Santamaría, Ricardo Díaz-Delgado, Javier Bustamante and Rocío Márquez-Ferrando.

### Study area description

Doñana LTSER (Long-Term Socio-Ecological Research) Platform. Doñana Protected Area. Doñana National and Natural Park. Doñana Biological Reserve (RBD).

### Design description

The dataset presented here belongs to three different projects (eLTER, ICTS-Doñana and LIFE Adaptamed) and can be divided into two periods (2011–2016 and 2017–2021) due to the addition of new sites in 2017 and the application of vegetation treatments since 2019 in the study area. The eLTER project focuses on facilitating long-term data exchange between European research infrastructure on climate change, biodiversity loss, soil degradation, pollution and unsustainable resources in European ecosystems. The aim is to improve the ability to predict long-term trends or responses of ecosystems to multiple pressures at local, regional and continental scales. Doñana LTSER platform will share long-term data on biodiversity and environmental processes. Long-term monitoring of small mammals in Doñana, as part of the eLTER project, started in 2011 in Matasgordas, an area of high ecological value in DNP. In 2017, the monitoring was incorporated into the ICTS-Doñana project as part of its programme to assess the dynamics of natural processes, species and communities since the 1970s. The initiative generates time-series data used to analyse long-term trends and evaluate changes in Doñana’s conservation status in response to climate and global change. Additionally, small mammals were also considered within the framework of the LIFE Adaptamed project in the same year. This project aims to increase the resilience of ecosystem services to the effects of climate change using ecosystem-based adaptation (EbA) (Barea-Azcón et al. 2022). For this purpose, a habitat management experiment with different vegetation treatments was designed and small mammals surveys included. The information obtained was incorporated into the long-term monitoring dataset already in place.

Therefore, this dataset provides valuable information to detect changes in biodiversity and ecological functioning over time in Doñana, which could be used in conservation and management plans.

### Funding

The project has benefitted from the eLTER Plus INFRAIA research project (Horizon 2020 EU programme, agreement no. 871128), the eLTER H2020 INFRAIA project (Horizon 2020 EU programme, agreement no. 654359) and NextGenerationEU funding. We also acknowledge the financial support of the Singular Scientific and Technical Infrastructures of the Spanish Ministry of Science and Innovation (ICTS-MICINN), the Ministry of Agriculture, Livestock, Fisheries and Sustainable Development of the Government of Andalusia (CAGPDES-JA), the Doñana Biological Station of the Spanish National Research Council (EBD-CSIC) providing in-kind and direct funding to maintain the programme, the Ministry of Environmental Sustainability and the Blue Economy of the Regional Government of Andalusia since 2017 with the LIFE-ADAPTAMED project and the Ministry of Science and Innovation (Recovery, Transformation and Resilience Plan).

## Sampling methods

### Study extent

The study area is located in south-western Spain, in the Guadalquivir Basin and includes the Natural Park of 682.236 km^2^ and the National Park of 542.51 km^2^ (Fig. 1). The climate is Mediterranean subhumid with Atlantic coastal influence: wet mild winters and dry warm summers. The rainy season is between October and April, with a peak in December–January (average annual rainfall is about 550 mm). Four main ecosystems are described in the area: temporary marshes, mobile dunes, Mediterranean scrubland with pine forests and sandy dunes stretching over a 30 km coastline.

### Sampling description

**Figure 2.**
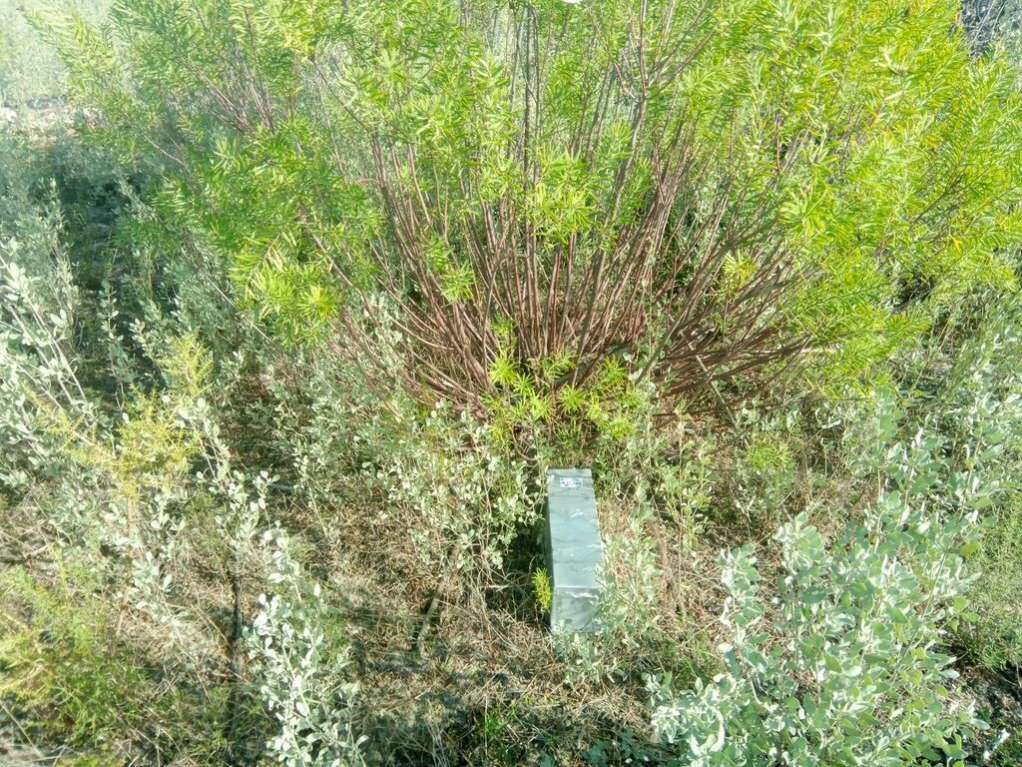
A Sherman trap used for the live capture of small mammals in Doñana (Credit: Francisco Carro).

To record the abundance and distribution of species, Sherman live traps (Fig. 2) were used for capture and recapture individuals following the methodology described in SEMICE (SEguimiento de MIcromamíferos Comunes de España) project for monitoring small mammals in the Mediterranean Region (https://www.semice.org/es/el-proyecto/; Flowerdew et al. (2004), Carro et al. (2023)). Five species were captured (see the taxonomic coverage section below).

**Figure 3.**
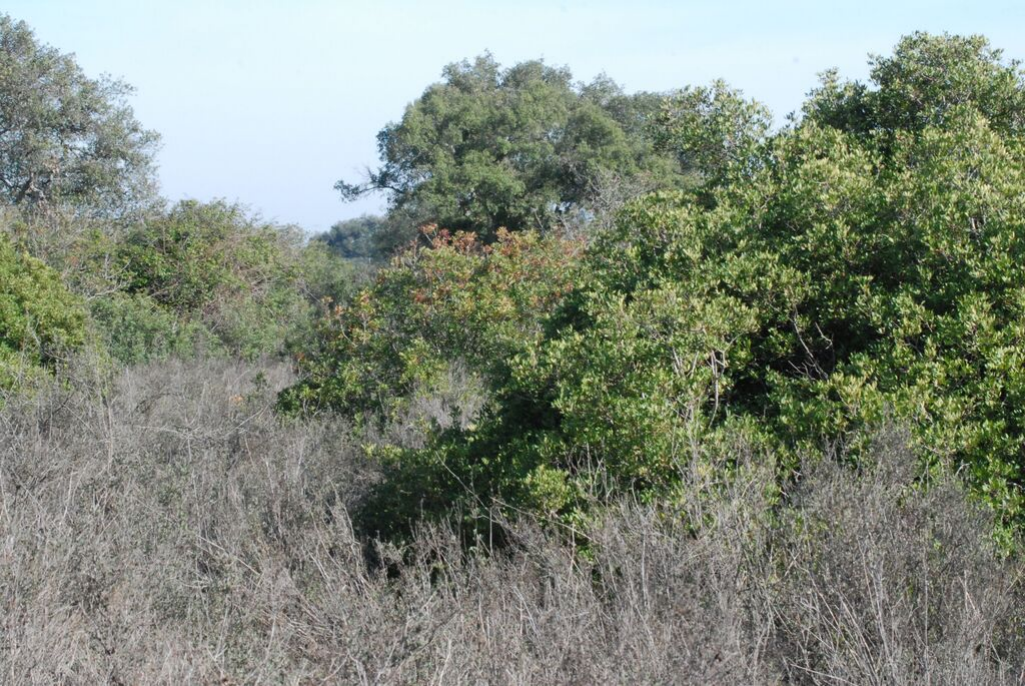
Mediterranean cork oak (*Quercus suber*) forest with hygrophytic Atlantic scrubs (*Chamaerops humilis* and *Pistacia lentiscus*), representative vegetation of Matasgordas 3D sampling site (Credit: Francisco Carro).

**Figure 4.**
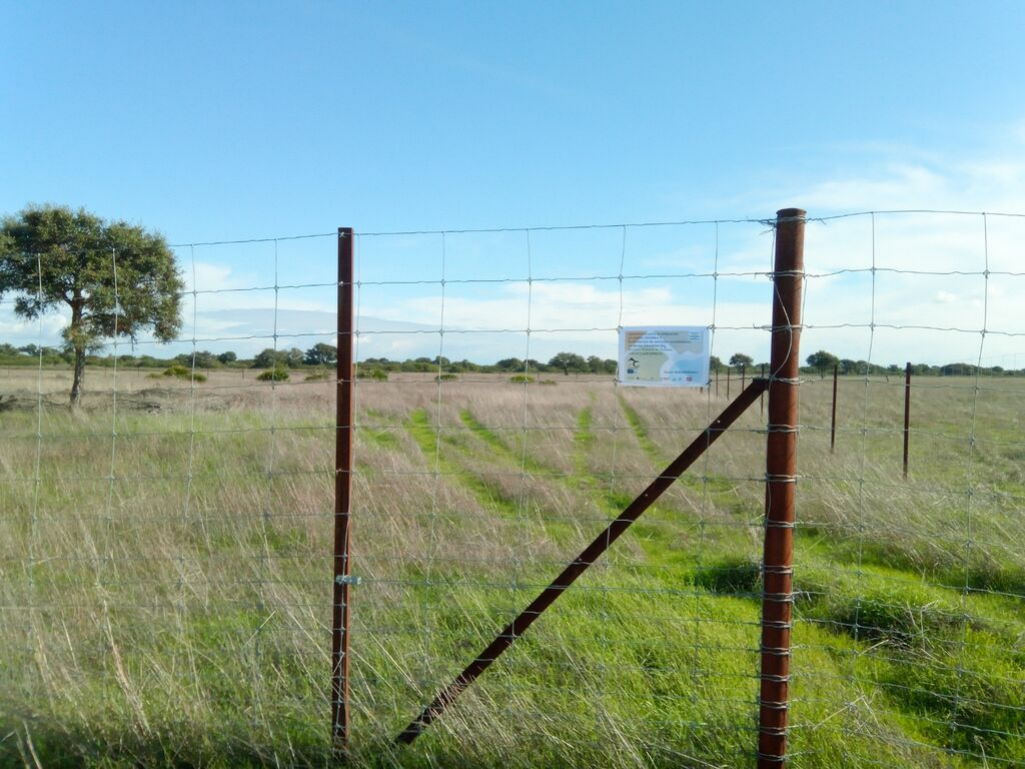
Matasgordas 3B sampling site, enclosed by fencing to exclude ungulate grazers. (Credit: Xosé Pardavila).

**Figure 5.**
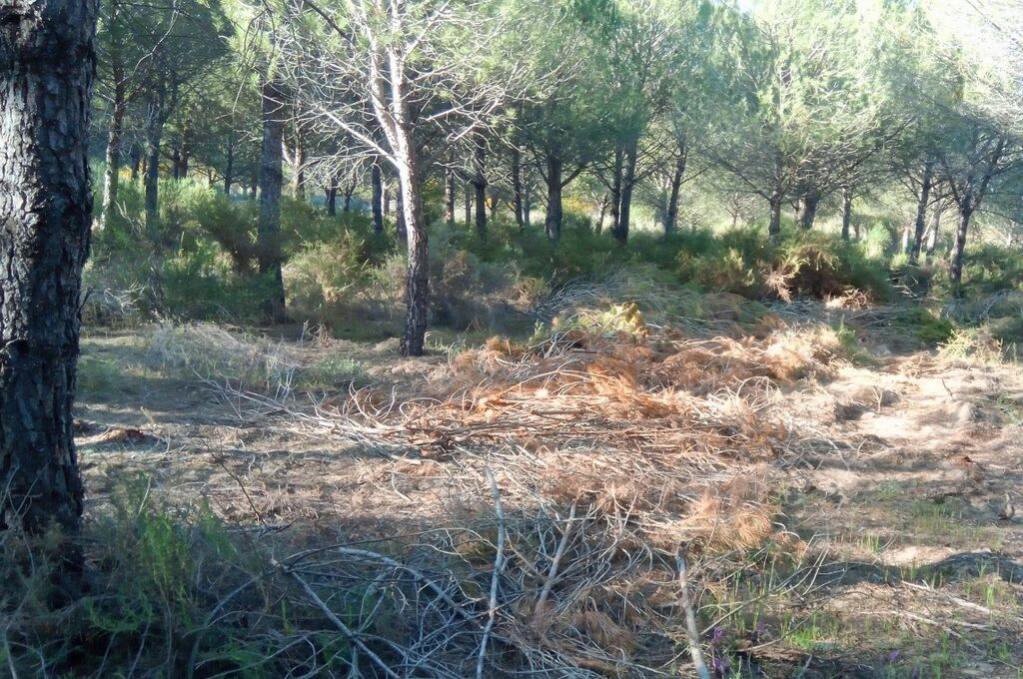
Mediana 2D sampling site, characterised by pine trees and xerophytic Mediterranean scrub, underwent a thinning treatment that reduced 60% of the vegetation (Credit: Xosé Pardavila).

Sampling was conducted in Matasgordas (2011-2021), an area of high ecological value within Doñana National Park (DNP), as well as in five additional localities (2017-2021): two within the DNP (Manecorro and Sabinar) and three within the Natural Park (Mediana, Moro and Loro) (Fig. 1). These sites differ in vegetation type — including scrubland, grassland, pine forest, oak forestand juniper forest — and in species composition (Figs 3, 4, 5).

The localities are seasonally sampled twice a year (spring and autumn). Each site — Manecorro, Matasgordas (Fig. 3), Mediana, Moro, Loro and Sabinar — was labelled by the first three letters of its name and divided into plots (1, 2, 3, 4, 5 or 6) and subplots (A, B, C or D): MAN3 (A, B, C, D), MAT3 (B, C, D), MAT4 (A), MAT5 (A), MED2 (B, C, D), MED3 (A, C, D), MOR1 (B, D), MOR2 (A, C), LOR1 (A, B, D), LOR2 (A, B, C), LOR4 (B, C, D), LOR6 (A, C, D), SAB1 (A), SAB2 (A) and SAB3 (A). To assess ecosystem resilience to the effects of climate change, a variety of vegetation treatments were implemented in the subplots. These treatments included fencing to reduce herbivore activity by excluding them (Fig. 4), cutting to thin vegetation by 60% (Fig. 5) and planting to establish fertility islands by sowing seeds and planting seedlings of cork oak (*Quercus suber*), lentisk (*Pistacia lentiscus*) and olive trees (*Olea europaea*). The plantation was protected with or without branches or a mix of both, serving as nursery structures. Other plots remained untreated as controls. The combinations of these treatments applied across sampling sites and dates are summarised in Table 1. Additionally, LOR1 and LOR2 were affected by a wildfire in June 2017.

### Quality control

Data quality control is essential to provide accurate and precise environmental information. We use several quality control mechanisms that allow us to automatically maintain information at a high-quality level. During the initial survey, the coordinates of subplots are recorded with GPS to serve as references for subsequent visits. In the field, data were digitally gathered using a CyberTracker sequence on mobile devices, where a step-by-step screen flow minimises the risk of data loss. Additionally, maximum and minimum thresholds for various variables are applied, automatically enhancing data quality and reducing errors. The collected data were then uploaded to a central CyberTracker database for preliminary quality assessment, with further validation conducted promptly in the office. Although several technicians participated in the data collection process, two highly skilled team members (FC and XP) consistently managed taxonomic identification, markings and body mass measurements, ensuring accuracy in these critical tasks. The capture of small mammal was carried out in agreement with the Animal Experimentation Committee of the Spanish National Research Council (CSIC) and the Ministry of Agriculture, Livestock, Fisheries and Sustainable Development, Junta de Andalucía (19/03/2017/133).

### Step description

A 6 × 6 grid of 36 traps, spaced 15 m apart, was arranged at each sampling site (subplot), covering an area of 0.56 hectares. To maintain statistical independence and avoid pseudo-replication, the distance between sampling sites was set to over 200 m. Sherman traps, measuring 10.16 × 11.43 × 38.1 cm, were used to capture small mammals. Each trap was numbered, positioned near bushes and set in the morning to remain active for three consecutive nights (72 hours). Each trap-night (one trap set for one night) was considered as a sampling event in the dataset (see data resources section below). This provided a number of captures per trap-night. Trap-nights were used to quantify sampling effort. Additionally, the traps were baited with bread soaked in olive oil and mealworm (*Tenebrio molitor*) larvae; hence, they were lined with synthetic cotton for insulation along with plastic film to protect them against adverse weather conditions. Traps were checked daily in the mornings during the sampling period. Night checks were deemed unnecessary due to the low abundance of shrews, which are capable of feeding on mealworm larvae to survive.

Traits data collection (see "Traits coverage" section below) for larger individuals, such as rats and dormice, were more challenging due to handling difficulties and therefore required anaesthesia via inhalation of 5% isoflurane. The anaesthetic was administered using a sedation hood, where individuals remained for less than one minute before data collection. Since 2017, to monitor recaptures of rats and dormice, trapped animals were marked with an ear tag displaying a numerical code at the base of the ear. Shrews were temporarily marked or released without undergoing standard procedures due to their high metabolism and current risk of mortality. All animals were released at the same site where they were captured. The trapping method remained consistent throughout the entire study period.

**Figure 6.**
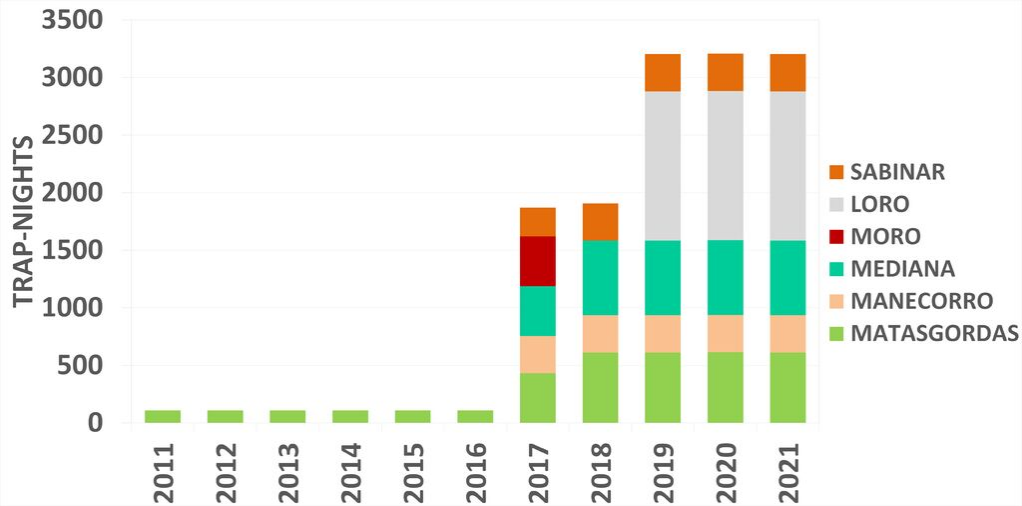
Trap-nights per locality for the autumn during the study period (2011-2021) in Doñana.

**Figure 7.**
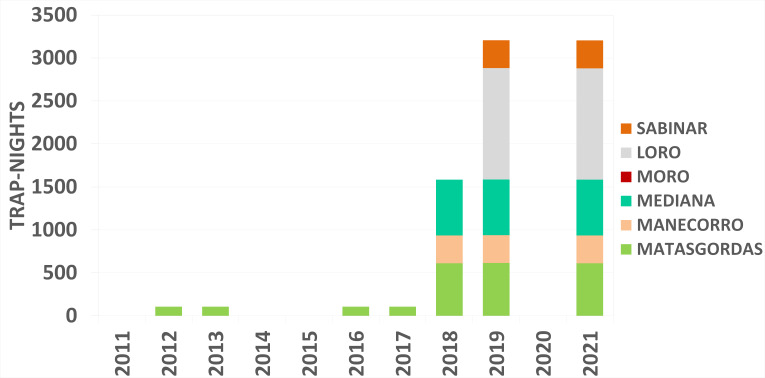
Trap-nights per locality for the spring during the study period (2011-2021) in Doñana.

**Figure 8.**
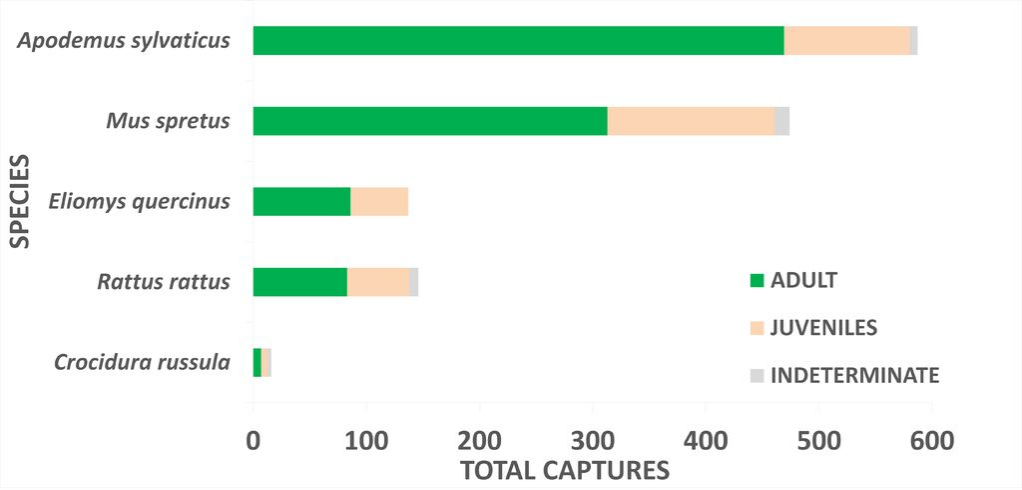
Total number of captures by age class of small-mammal species trapped during the study period (2011-2021) in Doñana.

Over the ten-year period, the sampling effort resulted on 22,470 trap-nights (Figs 6, 7) with 1,360 captures yield on: 587 captures (including 142 recaptures) of *A. sylvaticus*, 474 (102) of *M. spretus*, 137 (37) of *E. quercinus*, 146 (46) of *R. rattus* and 16 (0) of *C. russula* (Fig. 8). The number of set trap-nights and the abundance estimates for each species, categorised by sampling year and season, are shown in Table 2.

## Geographic coverage

### Description

The six localities included in this monitoring study are located across the Doñana National and Natural Park (Fig. 1). These plots are included in Doñana LTSER Platform, which, in turn, fall within the Doñana Protected Area.

### Coordinates

36.79 and 37.34 Latitude; −6.16 and −6.80 Longitude.

## Taxonomic coverage

### Description

**Figure 9.**
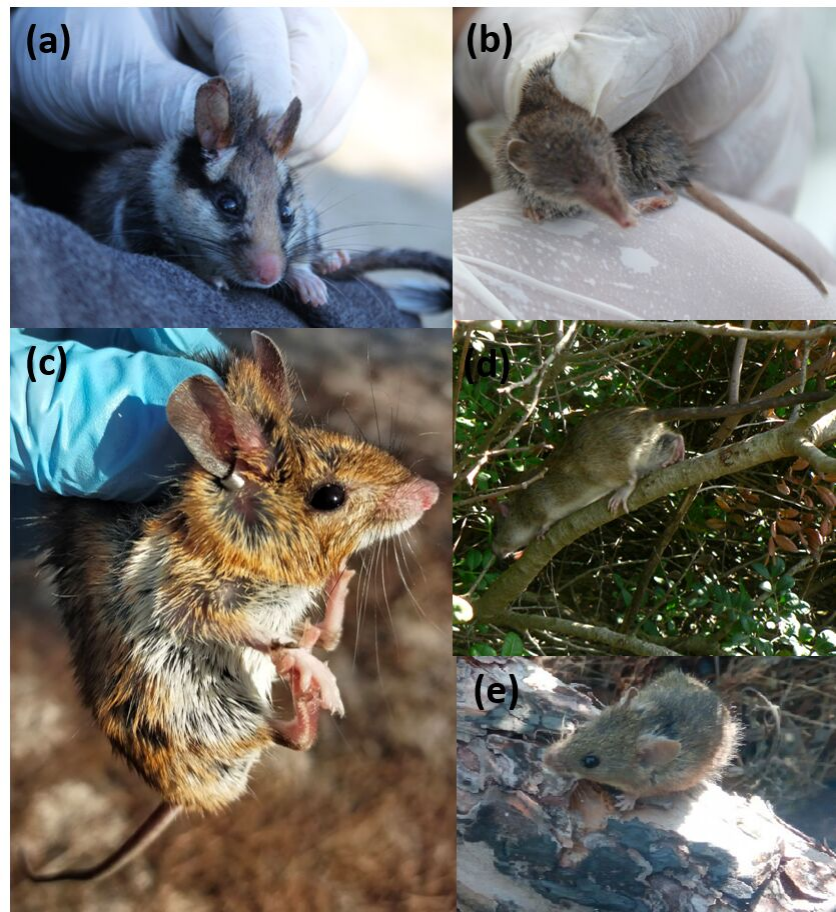
Small mammals species captured in the study area during the sampling period (2011-2021) in Doñana: (a) *Eliomys quercinus*; (b) *Crocidura russula*; (c) *Apodemus sylvaticus*; (d) *Rattus rattus* (Credit: Francisco Carro); and (e) *Mus spretus* (Credit: Xosé Pardavila).

Throughout the monitoring period, five small-mammal species, belonging to two orders and three families, were captured and recorded: garden dormouse, white-toothed shrew, wood mouse, black rat and Algerian mouse (Fig. 9). The most abundant species was *A. sylvaticus*, accounting for 43.16% of the total captures, while the least abundant was *C. russula*, representing only 1.18% of the total captures (Fig. 8).

### Taxa included

**Table:** 

Rank	Scientific Name	Common Name
species	*Crocidura russula*	White-toothed shrew
species	*Rattus rattus*	Black rat
species	*Eliomys quercinus*	Garden dormouse
species	*Mus spretus*	Algerian mouse
species	*Apodemus sylvaticus*	Wood mouse

## Traits coverage

Once captured, individuals were identified to the species level. Traits such as age (juvenile or adult) and sex (male or female) were recorded, when possible. Body mass was measured using a Pesola scale with an accuracy of 0.5 g. This information improves our knowledge of the population dynamics in small mammal species, which respond rapidly to environmental changes in a short period of time.

## Temporal coverage

### Notes

From 2011-08-11 to 2021-11-30.

## Usage licence

### Usage licence

Other

### IP rights notes

This work is licensed under a Creative Commons Attribution (CC-BY 4.0) License.

## Data resources

### Data package title

Long-term monitoring of small mammals (abundance and distribution) in Doñana Natural Area 2011–2021.

### Resource link

https://doi.org/10.15470/d0uktr

### Alternative identifiers

https://www.gbif.org/dataset/375567b6-a497-4beb-b093-44e69cf7a2e8

### Number of data sets

1

### Data set 1.

#### Data set name

Long-term monitoring of small mammals (abundance and distribution) in Doñana Natural Area 2011–2021.

#### Data format

Darwin Core.

#### Download URL

https://ipt.gbif.es/archive.do?r=don_icts-rbd_smallma_2011-2021&v=1.15

#### Description

The dataset comprises three interconnected tables stored as text files: sampling events (Event Core) (Figs 6, 7), occurrences (Occurrence Extension) and an extended Measurement or Fact extension (MoF) (Carro et al. 2025). These tables document the abundance and distribution of small mammals in Doñana from 2011 to 2021 across six localities within the National and Natural Park. The sampling event data represents a trap-night event. The Occurrence Extension includes additional details, such as the age class (adult or juvenile) and sex (female or male) of the captured individuals. Meanwhile, the MoF table contains body mass data for the individuals.

**Data set 1.** 

Column label	Column description
id (Event core, Occurrence extension, MoF)	Identifier of the sampling event.
parentEventID (Event core)	An identifier for the broader events that are grouped.
type (Event core)	The nature of a record.
licence (Event core)	Licence of dataset.
institutionID (Event core)	An identifier for the institution having custody of the information referred to in the record.
datasetID (Event core)	Identifier of the dataset including DOI.
institutionCode (Event core)	The name (or acronym) in use by the institution having custody of the object(s) or information referred to in the record.
datasetName (Event core)	Name of the published dataset.
samplingProtocol (Event core)	The references to the protocol used for the event.
sampleSizeValue (Event core)	The numeric value for a measurement of the size of the sample in an event (length of the transect or the area of a plot).
sampleSizeUnit (Event core)	The unit of measurement of the size of the sample in an event.
samplingEffort (Event core)	The amount of effort expended during the event.
eventDate (Event core)	The date during which the event occurred.
year (Event core)	The year during which the event occurred.
month (Event core)	The month during which the event occurred.
day (Event core)	The day during which the event occurred.
habitat (Event core)	A category or description of the habitat in which the eventID occurred.
continent (Event core)	The name of the continent in which the location occurs.
country (Event core)	The name of the country in which the location occurs.
countryCode (Event core)	The standard code for the country in which the location occurs.
stateProvince (Event core)	The name of the province in which the location occurs.
county (Event core)	The name of the county in which the location occurs.
municipality (Event core)	The name of the municipality in which the location occurs.
locality (Event core)	Specific description of the location of the study sites.
locationID (Event core)	An identifier for the location.
minimumElevationInMetres (Event core)	The lower altitude above sea level in metres.
maximumElevationInMetres (Event core)	The higher altitude above sea level in metres.
verbatimElevation (Event core)	The original altitude above sea level of the Location.
decimalLatitude (Event core)	The geographic latitude (in decimal degrees) of the geographic centre of the sampling plot.
decimalLongitude (Event core)	The geographic longitude (in decimal degrees) of the geographic centre of the sampling plot.
geodeticDatum (Event core)	The geodetic datum upon which the geographic coordinates given in decimal Latitude and decimal Longitude are based.
coordinateUncertaintyInMetres (Event core)	The horizontal distance (in metres) from the given decimal Latitude and decimal Longitude describing the smallest circle containing the whole of the Location.
eventRemarks (Event core)	Comments or notes about the event.
modified (Occurrence extension)	Date of modification.
language (Ocurrence extension)	Language of dataset.
collectionCode (Occurrence extension)	Code of the monitoring collection.
basisOfRecord (Occurrence extension)	Method of species identification.
dynamicProperties (Occurrence extension)	Additional measurements, facts, characteristics or assertions about the record. In this case, an indication of whether an individual was captured for the first time or it was recaptured.
occurrenceID (Occurrence extension)	Identifier for the occurrence.
recordedBy (Occurrence extension)	Names of observers responsible for recording the original occurrence.
individualCount (Occurrence extension)	The number of individuals present at the time of the occurrence.
lifeStage (Occurrence extension)	Age class of the individual recorded at the time of the occurrence.
sex (Occurrence extension)	The sex of the individual recorded in the occurrence.
identifiedBy (Occurrence extension)	Name or names of the Observer/s identifying the taxon.
scientificName (Occurrence extension)	Species scientific name.
kingdom (Occurrence extension)	Kingdom of the species.
phylum (Occurrence extension)	Taxonomic Phylum of the species.
class (Occurrence extension)	Taxonomic Class of the species.
order (Occurrence extension)	Taxonomic Order of the species.
family (Occurrence extension)	Taxonomic Family of the species.
genus (Occurrence extension)	Taxonomic Genus of the species.
specificEpithet (Occurrence extension)	Taxonomic Epithet of the species.
taxonRank (Occurrence extension)	Taxonomic Rank of the identification.
scientificNameAuthorship (Occurrence extension)	Authorship of scientific name.
occurrenceRemarks (Occurrence extension)	Comments or notes about the occurrences.
occurrenceStatus (Occurrence extention)	A statement about the presence or absence of a taxon at a Location.
organismQuantity (Ocurrence extention)	A number or enumeration value for the quantity of organisms.
organismQuantityType (Occurrence extention)	The type of quantification system used for the quantity of organisms.
measurementID (MoF)	Identifier for the measurement.
measurementType (MoF)	The nature of the measurement.
measurementValue (MoF)	The value of the measurement.
measurementAccuracy (MoF)	The potential error associated with the measurement value. The nature of the measurement.
measurementUnit (MoF)	The unit of the measurement value.
measurementDeterminedBy (MoF)	Names of observers who determined the value of the measurement.
measurementMethod	Description of the method used to determine the measurement.

## Figures and Tables

**Table 1.** Vegetation treatments and their application dates at each study site during the period of 2018– 2021.

**Plots**	**Subplots**	**Oct 2018**- **Feb 2019**	**Nov 2019-Jan 2020**	**Feb 2020-Mar 2020**	**Apr 2021- May 2021**
Matasgordas3	B		Fencing	Planting	
Matasgordas3	C		Fencing	Planting	
Matasgordas3	D				
Matasgordas4	A				
Matasgordas5	A				
Manecorro3	A		Fencing	Planting	
Manecorro3	B				
Manecorro3	C			Planting	
Manecorro3	D				
Mediana2	B	Cutting	Fencing	Planting	
Mediana2	C	Cutting		Planting	
Mediana2	D	Cutting			
Mediana3	A			Planting	
Mediana3	C				
Mediana3	D		Fencing	Planting	
Loro1	A				Cutting Planting
Loro1	B				Cutting Fencing Planting
Loro1	D				Cutting
Loro2	A				Planting
Loro2	B				Fencing Planting
Loro2	C				Planting
Loro4	B, C and D				
Loro6	A, C and D				
Sabinar1	A				
Sabinar2	A				
Sabinar3	A				
Moro1	B and D				
Moro2	A and C				

**Table 2.** Trap-nights, relative abundance (number of individuals caught/100 trap-night) and absolute abundance (number of individuals caught in parenthesis) of each species by sampling year and season.

Year	Season	Trap-nights	*A. sylvaticus*	*M. spretus*	*E. quercinus*	*R. rattus*	*C. russula*
2011	Spring	-	-	-	-	-	-
	Autumn	108	0	3.70 (4)	0	3.70 (4)	0
2012	Spring	108	14.81 (16)	0	0	9.30 (10)	0
	Autumn	108	1.85 (2)	0.93 (1)	0	4.63 (5)	0
2013	Spring	108	1.85 (2)	0	0	0	0
	Autumn	108	0.93 (1)	21.30 (23)	0	0.93 (1)	0
2014	Spring	-	-	-	-	-	-
	Autumn	108	5.56 (6)	23.15 (25)	0	7.41 (8)	0
2015	Spring	-	-	-	-	-	-
	Autumn	108	0.93 (1)	8.33 (9)	0.93 (1)	4.63 (5)	0
2016	Spring	108	0.93 (1)	0	0.93 (1)	0	0
	Autumn	108	0	4.63 (5)	0	0	0
2017	Spring	108	2.78 (3)	0	0	0	0
	Autumn	1872	0.53 (10)	0.64 (12)	0	0.21 (4)	0
2018	Spring	1584	2.02 (32)	0.13 (2)	0.38 (6)	0.95 (15)	0.19 (3)
	Autumn	1908	1.57 (30)	10.90 (208)	0.52 (10)	0.68 (13)	0.16 (3)
2019	Spring	3207	4.08 (131)	0.37 (12)	0.87 (28)	0.81 (26)	0.03 (1)
	Autumn	3204	2.62 (84)	1.03 (33)	0.50 (16)	0.56 (18)	0.12 (4)
2020	Spring	-	-	-	-	-	-
	Autumn	3207	1.71 (55)	2.71(87)	0.12 (4)	0.16 (5)	0.06 (2)
2021	Spring	3204	2.75 (88)	0.37 (12)	1.72 (55)	0.50 (16)	0.03 (1)
	Autumn	3204	3.9 (125)	1.28 (41)	0.50 (16)	0.50 (16)	0.06 (2)
